# Nanoscale Kerr Nonlinearity Enhancement Using Spontaneously Generated Coherence in Plasmonic Nanocavity

**DOI:** 10.1038/srep18315

**Published:** 2015-12-16

**Authors:** Hongyi Chen, Juanjuan Ren, Ying Gu, Dongxing Zhao, Junxiang Zhang, Qihuang Gong

**Affiliations:** 1 State Key Laboratory for Mesoscopic Physics, Collaborative Innovation Center of Quantum Matter, School of Physics, Peking University, Beijing, 100871, China; 2 Collaborative Innovation Center of Extreme Optics, Shanxi University, Taiyuan, Shanxi, 030006, China; 3 State Key Laboratory of Quantum Optics and Quantum Optics Devices, Institute of Opto-Electronics, Shanxi University, Taiyuan, Shanxi 030006, China

## Abstract

The enhancement of the optical nonlinear effects at nanoscale is important in the on-chip optical information processing. We theoretically propose the mechanism of the great Kerr nonlinearity enhancement by using anisotropic Purcell factors in a double-Λ type four-level system, i.e., if the bisector of the two vertical dipole moments lies in the small/large Purcell factor axis in the space, the Kerr nonlinearity will be enhanced/decreased due to the spontaneously generated coherence accordingly. Besides, when the two dipole moments are parallel, the extremely large Kerr nonlinearity increase appears, which comes from the double population trapping. Using the custom-designed resonant plasmonic nanostructure which gives an anisotropic Purcell factor environment, we demonstrate the effective nanoscale control of the Kerr nonlinearity. Such controllable Kerr nonlinearity may be realized by the state-of-the-art nanotechnics and it may have potential applications in on-chip photonic nonlinear devices.

Conventional nonlinear effects in bulk materials restricted their applications in realizing on-chip optical information processing. Plasmonic nanostructure becomes one of the competitive nanoscale platforms to demonstrate nonlinear optical effects^1^,^2^. Originating from the free electrons collective oscillation^3^, the surface plasmons have the ability to confine the electromagnetic field into an extremely small mode volume, thus leading to a large enhancement of the near field^4^,^5^,^6^,^7^,^8^,^9^,^10^. Based on this property, various nonlinear optical effects have been investigated theoretically and experimentally^11^,^12^,^13^,^14^,^15^,^16^,^17^,^18^,^19^,^20^,^21^. While, another key advantage of the plasmonic structure is the large subwavelength scale anisotropic Purcell factors^22^,^23^, which have been widely used in the linear quantum optical effects, such as the enhancement and quenching of molecular fluorescence^24^,^25^,^26^,^27^,^28^, double coherent population trapping^29^, and modification of the spontaneous emission spectrum^30^. Though, the enhanced nonlinear susceptibilities^31^ and nonlinear optical rectification^32^ due to the suppression of spontaneous emission induced by surface plasmons have been reported. However, the study of the nonlinear optical effects with the help of the anisotropic Purcell factors is still rare.

The Kerr-type nonlinearity, known as one of the most fundamental coefficients in nonlinear optics, corresponds to the refractive part of the third-order susceptibility of optical media. It plays a crucial role in the cross-phase modulation for quantum logic operations^33^, modulation for generation of optical solitons^34^,^35^, superposition states for quantum information processing^36^, etc. Various types of the methods using quantum coherence to enhance the Kerr nonlinearity have been presented^37^,^38^,^39^,^40^,^41^,^42^. Based on electromagnetically induced transparency (EIT), the Kerr nonlinearity is greatly enhanced near the two-photon resonance in conventional three-level atomic system^37^. Subsequently, for four-level Rubidium atomic system, several orders of magnitude greater than the Kerr nonlinearity of three-level scheme was observed^38^. Because of the interaction of double dark resonances, giant enhancement of the Kerr nonlinearity was proposed^39^. In addition, the influence of spontaneously generated coherence (SGC)^43^ on the enhanced Kerr nonlinearity was also investigated in three-level atomic system^40^. It is found that the SGC plays a role only when the dipole moments are nonorthogonal in vacuum. However, in natural atoms, the dipole moments between the two near-degenerate energy levels are generally vertical, which limited its experimental realization of the Kerr nonlinearity enhancement based on the SGC. Here, we use the SGC to modify the Kerr nonlinearity of an EIT-like system via plasmonic nanocavity. With even vertical dipole moments, the SGC can still take into effect in the anisotropic Purcell factors.

In the following, letting all the transition channels influenced by the well-designed plasmonic nanocavity, we first theoretically demonstrate the mechanism of the great Kerr nonlinearity enhancement by using anisotropic Purcell factors in a double-Λ type four-level system. If the bisector of the two vertical dipole moments lies in the small/large Purcell factor axis, the Kerr nonlinearity will be enhanced/decreased due to the SGC accordingly. What’s more, we find that the Kerr nonlinearity could be further increased by adjusting the atomic energy level spacing, detuning and Rabi frequency of the coherent field. If the dipole moments are parallel, an extremely large Kerr nonlinearity in the middle peak is achieved under the double trapping condition. Using the custom-designed plasmon nanocavity, we can control the Kerr nonlinearity at the subwavelength scale due to the fact that Kerr nonlinearity is very sensitive to the positions. This hybrid system may offer the better understanding of the quantum light-matter interaction at nanoscale and the potential application in ultra-compact optoelectronic quantum nonlinear devices.

## Results

### Model Setup

**Figure 1:**
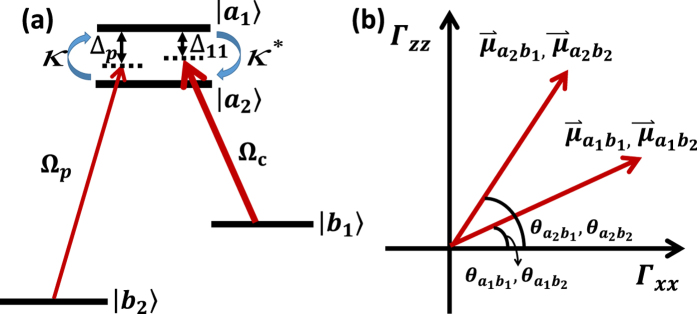
The schematic of the double-Λ type four-level atomic system. (**a**) The schematic of a four-level double-Λ type atomic system with the cross damping *κ* (κ^*^) between the two upper near-degenerate levels. (**b**) The dipole moments of all the related channels. Γ_*xx*_ (Γ_*zz*_) denotes the decay rate along the *x* (*z*) direction. 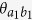, 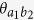 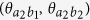 are the angles between the dipole moments 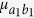, 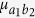 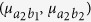 and the *x* axis.

As shown in Fig. 1, a double-Λ type four-level atomic system is considered, which consists of two near-degenerate upper levels 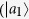 and 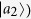 and two lower levels 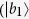 and 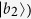. Due to the two closely lying upper levels, a strong field with the frequency *ν*_1_ simultaneously pumps the transitions between 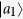, 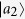 and the lower state 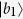, and a weak field with the frequency *ν*_2_ simultaneously probes the transitions between 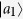, 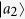 and the lower state 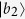. The optical frequencies corresponding to four levels are 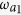, 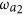, 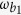, and 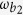, so the optical detunings and two upper levels energy spacing are 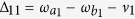, 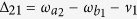, 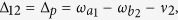 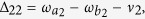 and 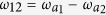. Our model is based on the typical EIT structure configuration with all the transition channels are coupled by optical fields. Under the Weisskopf-Wigner approximation, the spontaneous decay rate from the upper level 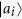 to the lower level 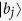 is defined as *γ*_*ij*_, *i*, *j* = 1, 2. Particularly, because the upper levels are near-degenerate, so their transition channels associated with same lower states will interact with the common vacuum mode. Thus the crossing damping between two upper levels exists and it is denoted by *κ*, κ^*^, where 
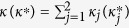
. Note that *κ*_1_ and *κ*_2_ are the contribution of the two upper levels interacting with 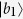 and 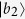, respectively.

Under the rotating-wave and dipole approximation, we can obtain the Hamitonian of the described system in the interaction picture^29^:


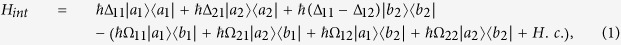


where 
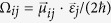
, for, *i*, *j* = 1, 2, is the Rabi frequency of the field pumping the transition between the upper level 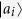 and the lower level 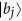. 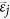 and 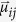 are the corresponding amplitude of the field and transition dipole moment. As shown later, the coherent field 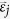 can be modified by a resonant plasmon structures at the nanoscale. Taking the two near-degenerate levels into account, we assume that the Ω_11_ = Ω_21_ = Ω_*c*_, Ω_12_ = Ω_22_ = Ω_*p*_, and all the dipole moments are equal, that is *μ*_11_ = *μ*_21_ = *μ*_12_ = *μ*_22_ = *μ*.

The master equation of the atomic system in the interaction picture is:


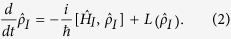


The first term is the interaction between the coupling filed and the atomic system. The second term is the dissipation term which reflects the effects of the environment to the system. Considering the characteristic in our system, the 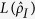 is given as 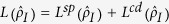, where 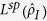 is the conventional spontaneous decay rates induced by the interaction of system with the vacuum modes and 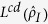 is the crossing damping rates between two upper levels. The expressions of the dissipation term are as follows:


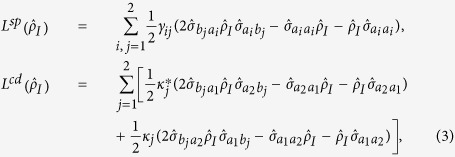


where 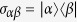, *α*, *β* = *a*_*i*_, *b*_*j*_ are the dipole transition operators, and *γ*_*ij*_ are the spontaneous decay rates between 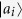 and 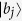. The detailed systematic equations of motion of the density matrix in the interaction picture (see equation (7) in Methods) gives the whole information about the effects of the interaction with the field and the environment to each density matrix element. From the expressions shown in Methods, it is obvious that the crossing damping vanishes when the dipole moments are vertical in isotropic vacuum, and the spontaneous decay rates *γ* and cross damping *κ* are connected to the decay rates along the *x* and *z* directions, that is Γ_*xx*_, Γ_*zz*_. In the following, we will see that the *x* and *z* directions have different Purcell factors Γ_*xx*_/*γ*_0_ and Γ_*zz*_/*γ*_0_, which could be realized in plasmonic nanocavity and it will guarantee that the crossing damping exists with the vertical dipole moments.

The response of the atomic medium is dominated by the intensity of polarization *P* = *ε*_0_(*ε*_2_*χe*^−*iωt*^ + *c*.*c*.)/2, where *ε*_2_ is the amplitude of the probe field, and *χ* is the susceptibility of the atomic medium. The expression of polarization in terms of dipole moment and density matrix can be obtained as 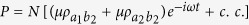 by performing a quantum average over the atomic ensemble of *N* atoms. The perturbation method^44^ is employed to get the steady-state solution of the equations of motion, which is essential for the derivation of the linear and nonlinear susceptibility. Then, the elements of the density matrix can be expanded as 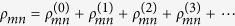. Assuming that the probe field is much weaker than the coupling field, we can find that the only nonzero density matrix element for the zeroth order is 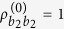. Using the perturbation method and under weak probe field limit, we obtain the elements of density matrix up to the third-order. With above results, the first-order and third-order susceptibilities *χ*^(1)^ and *χ*^(3)^ can be expressed as following:


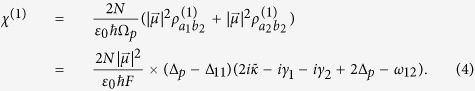



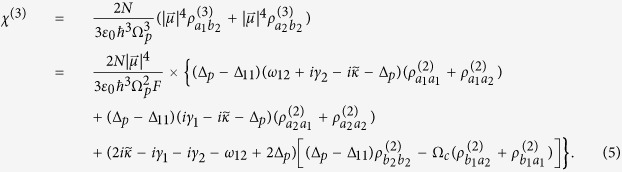


where 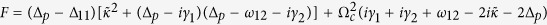,*γ*_11_ = *γ*_12_ = *γ*_1_, *γ*_21_ = *γ*_22_ = *γ*_2_, 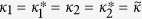, and *χ* is defined as


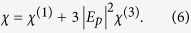


### Mechanism of Enhancing Kerr Nonlinearity in the Anisotropic Purcell Factors Space

**Figure 2:**
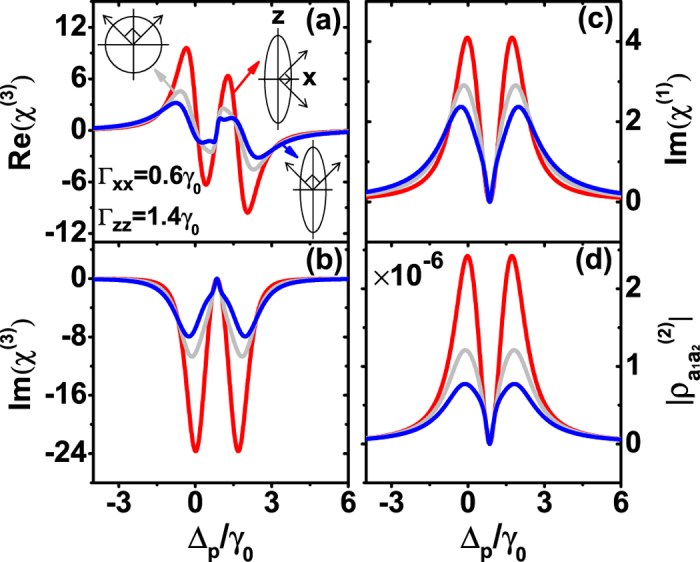
The mechanism of enhancing and suppressing the Kerr nonlinearity by anisotropic Purcell factors with orthogonal dipole moments. (**a**) Kerr nonlinearity Re(*χ*^(3)^), (**b**) nonlinear absorption Im(*χ*^(3)^), (**c**) linear absorption Im(*χ*^(1)^), and (**d**) coherence term 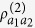 of the double-Λ type system as a function of probe detuning with different anisotropic Purcell factors and orthogonal dipole moments. Enhancing (red curve) or suppressing (blue curve) the Kerr nonlinearity occurs when the bisection of the two pairs of dipole moments lies along the axis of the small or large Purcell factor, compared with Kerr nonlinearity for the isotropic Purcell factor (grey curve). Parameters are *ω*_12_ = 1.7*γ*_0_, Δ_11_ = 0.85*γ*_0_, and Rabi frequencies Ω_*c*_ = 0.5*γ*_0_, Ω_*p*_ = 0.001*γ*_0_.

We begin by theoretically exploring the underlying mechanism of the anisotropic Purcell factors to modify the Kerr nonlinearity. According to the expressions (4) and (5), the numerical results of the refractive part of the third-order susceptibility, linear and nonlinear absorption, and coherence term 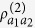 originated from SGC as a function of the probe detuning are displayed in Fig. 2. We normalize the *χ*^(1)^ and *χ*^(3)^ by the factor 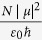 and 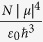, respectively. Here, the Purcell factors are set to be Γ_*xx*_ = 0.6*γ*_0_, Γ_*zz*_ = 1.4*γ*_0_, other parameters are *ω*_12_ = 1.7*γ*_0_, Δ_11_ = 0.85*γ*_0_, Ω_*c*_ = 0.5*γ*_0_, Ω_*p*_ = 0.001*γ*_0_. As shown in Fig. 2(a), if the angle bisector of two perpendicular dipole moments lies along the small Purcell factors axis (red curve) or lies on the large Purcell factors axis (blue curve), there is an enhancement or decrement of the Kerr nonlinearity occurred when compared with the isotropic vacuum with Γ_*xx*_ = Γ_*zz*_ = *γ*_0_ (gray curve). Owing to the anisotropic Purcell factors, the Kerr nonlinearity can be modified more effectively than the isotropic situation^42^ in double-Λ system with vertical dipole moments. The imaginary part of the third-order susceptibility in Fig. 2(b) is always negative which means a nonlinear gain. From Fig. 2(c), we can find that in the three cases, the linear absorption at the EIT point of Δ_*p*_/*γ*_0_ = 0.85 is vanishing in accord with the two-photon resonance Δ_*p*_ = Δ_11_ and the enhancement of the Kerr nonlinearity can be achieved near the transparent point.

Now we provide a qualitative explanation for the above numerical results. The coherence term 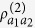 between the two upper near-degenerate levels is associated with the SGC and both the first-order term 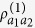 and third-order term 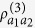 are found to be zero. Obviously, the enhancement (suppression) of the Kerr nonlinearity corresponds to the increasing (decreasing) of 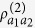 (Fig. 2(d)). Hence, we attribute the modification of Kerr nonlinearity to the SGC between the two upper near-degenerate levels. By properly choosing the anisotropic Purcell factors, the enhancement of the Kerr nonlinearity in our system could be easily realized.

**Figure 3:**
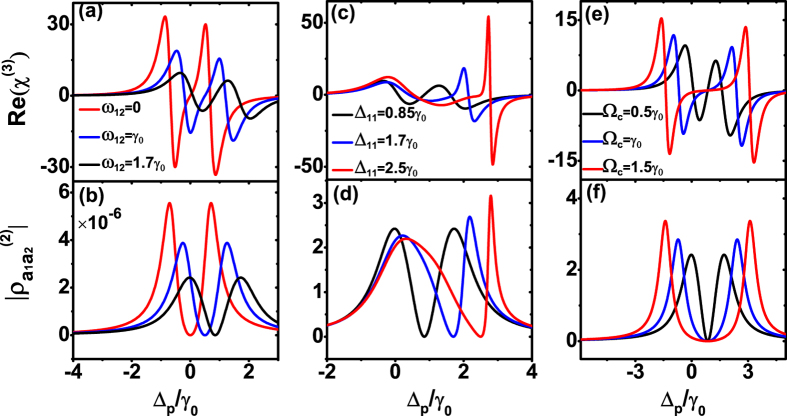
The modification of the Kerr nonlinearity by atomic and coupling filed parameters in orthogonal dipole moments. The Re(*χ*^(3)^) and 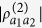 with varying (**a**,**b**) energy level spacing *ω*_12_, (**c**,**d**) coupling field detuning Δ_11_, and (**e**,**f**) coupling field Rabi frequency Ω_*c*_. Other parameters remain the same as in Fig. 2.

To fully investigate the modification of the Kerr nonlinearity, we numerically calculated the refractive part of the third-order susceptibility and the coherence term 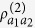 with different atomic energy level spacing *ω*_12_, the coupling field detuning Δ_11_ and Rabi frequency Ω_*c*_. The two pairs of the dipole moments are orthogonal and their bisections all lie along the *x* axis of decay rate with Γ_*xx*_ = 0.6*γ*_0_ and Γ_*zz*_ = 1.4*γ*_0_. As the energy level spacing *ω*_12_ decreases, the Kerr linearity increases as shown in Fig. 3(a). In this situation, the correspondence of the Kerr nonlinearity and the coherence term 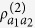 shows the origin of the enhanced Kerr nonlinearity can be traced to the SGC (Fig. 3(b)). Moreover, when the *ω*_12_ decreases from 1.7*γ*_0_ to 0 with the fixed detuning 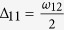 of the coupling field, the spectrum of the Kerr nonlinearity and the coherence term 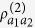 would shift accordingly with the EIT point. Further numerical calculations indicate that, as 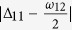 and the coupling Rabi frequency increase, the enhancement of the Kerr nonlinearity is obtained (Fig. 3(c,e)). The reason is also due to the SGC, i.e., the large values of the coherence term 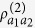 in Fig. 3(d,f) correspond to the peaks of Kerr nonlinearity in Fig. 3(c,e). As a reference, the parameters of the black curve in all the figures remain the same as in Fig. 2. Therefore, through the properly choosen *ω*_12_, Δ_11_ and Ω_*c*_, the Kerr nonlinearity could be further enhanced with the vertical dipole moments under anisotropic Purcell factors.

If the two pairs of the dipole moments are neither vertical nor parallel with each other, i.e., the angles between the two pairs dipole moments are either larger or smaller than 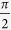, the conclusions remain the same. We attribute the enhancement of the Kerr nonlinearity to the increment of the 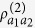 which originates from the SGC.

**Figure 4:**
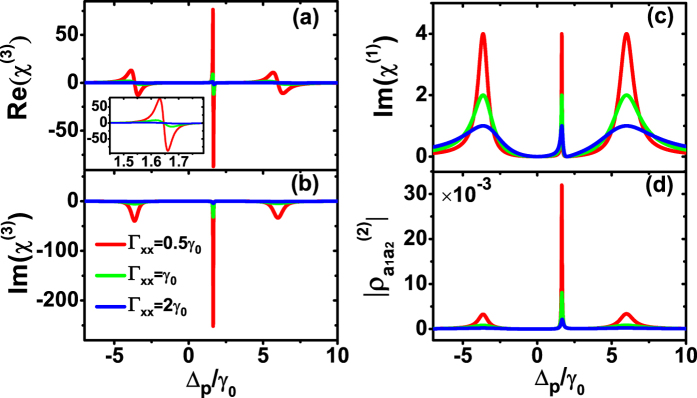
The mechanism of the Kerr nonlinearity enhancement by anisotropic Purcell factors in parallel dipole moments. (**a**) The Kerr nonlinearity Re(*χ*^(3)^), (**b**) nonlinear absorption Im(*χ*^(3)^), (**c**) linear absorption Im(*χ*^(1)^), and (**d**) coherence term 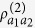 of the double-Λ type system with different anisotropic Purcell factors. The inset of (**a**) is the enlarged scale of the central peak. All the dipole moments lie on the *x* axis. The decay rates of the *z* direction all are set with Γ_*zz*_ = *γ*_0_. Other parameters are *ω*_12_ = 4*γ*_0_, Δ_11_ = 0, and Rabi frequencies Ω_*c*_ = 3*γ*_0_, Ω_*p*_ = 0.03*γ*_0_.

Next, we focus on the situation that the two pairs of the dipole moments are parallel. We first explore the mechanism of the anisotropic Purcell factors to modify the Kerr nonlinearity. In this system, letting the two pairs of the parallel dipole moments lie along the *x* axis, there is a double-EIT phenomenon induced by SGC, namely, one is the normal EIT which satisfies the two-photon resonance condition Δ_*p*_ = Δ_11_, the other is a kind of new transparency determined by the SGC^29^ (Fig. 4(c)). Although, the enhanced Kerr nonlinearity in the double-Λ system with the parallel dipole moments have been investigated^42^, the influence of the double-EIT to the Kerr nonlinearity is still unknown. By diagonalizing the Hamiltonian (equation (1)), the positions of absorption peaks are consistent with the prediction of dressed state analysis (Fig. 4(c)).

Comparing with the isotropic vacuum, the dipole moments lie on the small/large Purcell factors, the enhancing/decreasing of the Kerr nonlinearity happens (Fig. 4(a)), which is the same as found in the orthogonal dipole moments situation (Fig. 2(a)). But, different with the orthogonal situation, a huge enhancement in the central peak of the Kerr nonlinearity was found, which corresponds to the ultra narrow central peak in the linear absorption. Thus, the remarkably increase of coherence term 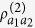 indicates that the SGC is the physical origin of the enhancing Kerr nonlinearity (Fig. 4(d)).

More numerical calculations indicate that, with the anisotropic Purcell factors, the Kerr nonlinearity would be further enhanced as the energy level spacing decreases and the coupling Rabi frequency grows. But when we study the effect of the different coupling field detunings on the Kerr nonlinearity, the results show that the closer to the 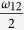 point, the bigger Kerr nonlinearity. Therefore, by properly adjusting the atom intrinsic parameter and the coupling field parameters, the Kerr nonlinearity could be further enhanced in the parallel dipole moments.

### The Nanoscale Realization of the Enhanced Kerr Nonlinearity in Plasmonic Structure

To achieve the Kerr nonlinearity enhancement at nanoscale, we propose a custom-designed hybrid system of the quantum emitter and the resonant plasmon nanostructure. Plasmonic nanocavity with the anisotropic local optical state density, which offers the subwavelength-confined anisotropic Purcell factor and strong near field, is a suitable candidate. Here, we use the Cesium atom hyperfine structure to represent the double-Λ type four-level system with orthogonal dipole moments. 6*D*_3/2_, *F* = 3 and 6*D*_3/2_, *F* = 2 correspond to the upper levels 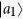 and 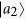, 6*P*_3/2_, and 6*P*_1/2_ are the two ground levels 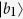 and 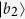, respectively. The upper levels to the lower level 6*P*_3/2_ have the transition wavelength of 920.85 nm, and the upper levels to the other lower level 6*P*_1/2_ have the transition wavelength of 876.14 nm. To demonstrate the mechanism mentioned above, we put a quantum system in the near field region of the plasmonic nanocavity.

**Figure 5:**
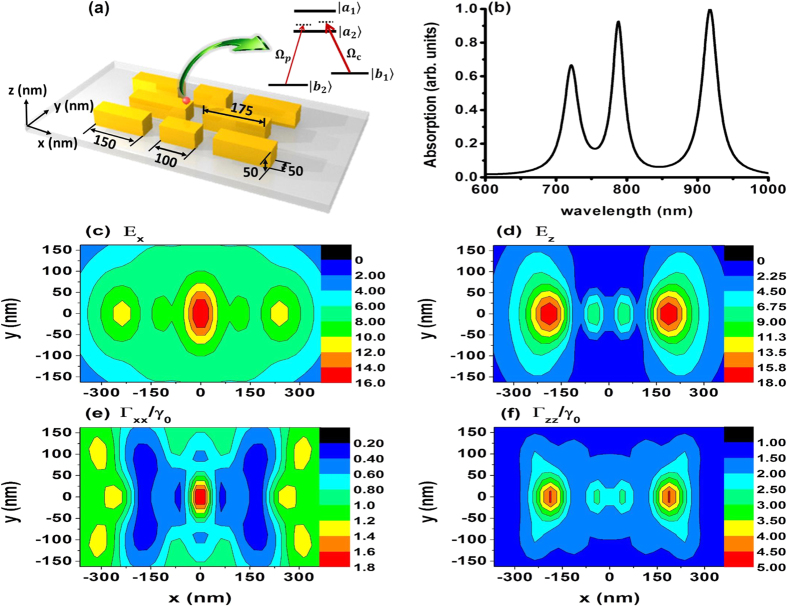
The resonant gold nanocavity with the near field and Purcell factor distributions. (**a**) Schematic of a resonant gold nanocavity to interact with the atom and (**b**) its absorption. The near field distributions (**c**) *E*_*x*_, (**d**) *E*_*z*_ and the distributions of anisotropic Purcell factors (**e**) Γ_*xx*_/*γ*_0_, (**f**) Γ_*zz*_/*γ*_0_ on the *xy*-plane 75 nm from the metallic surface and at the wavelength of *λ* = 920 nm (the origin of the coordinate is in the center point of the nanocavity).

In the following, using Green’s tensor method^45^,^46^ with the mesh of 25 nm, we design a gold nanocavity (Fig. 5(a)) composed of eight gold nanostrips with 50 nm spacing in both directions to support suitable Purcell factors. The two largest gold nanostripes with the size of 175 × 50 × 50 nm^3^ in the middle part of the nanocavity dominate the main resonance. Influenced by the other six nanostripes around it (more size details shown in Fig. 5(a)), the resonance wavelength of the nanocavity can be modified effectively and the region of the anisotropic Purcell factors can also be enlarged for better control of the Kerr nonlinearity. There are three peaks in the absorption spectrum of the nanocavity (Fig. 5(b)), among which, the largest one is the dipole resonance with the wavelength of *λ* = 917 nm, which matches the transitions from the upper levels 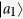 and 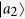 to the lower level 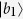. While, the probe field of *λ* = 876 nm is off resonance with the plasmonic nanocavity. Thus, the near field of the dipole resonance is strongly enhanced (Fig. 5(c,d)), which can make sure that the coupling field is strong enough compared with the off resonance probe field. Furthermore, the proposed plasmonic nanocavity structure guarantees the subwavelength scale Purcell factors. We then explored the decay rate distributions of the *xy* plane which is 75 nm away from the metallic surface at *λ* = 920 nm, and found that anisotropy in different positions is large enough for our investigation (Fig. 5(e,f)). For matching different transitions of the quantum emitter, the plasmonic nanostructure also can change its resonance wavelength by adjusting its structures, materials or dimensions, etc. As a simple example, this proposed design of the plasmonic nanocavity guarantees the suitable resonance wavelength, near field enhancement and the anisotropic Purcell factors for the study requirements. It offers the possibility to be a novel quantum nonlinear platform to realize controllable Kerr nonlinearity at nanoscale. Crucially, our gold nanostructure can be successfully fabricated in the laboratory with the help of the present state-of-the-art nanofabrication techniques^47^,^48^.

**Figure 6:**
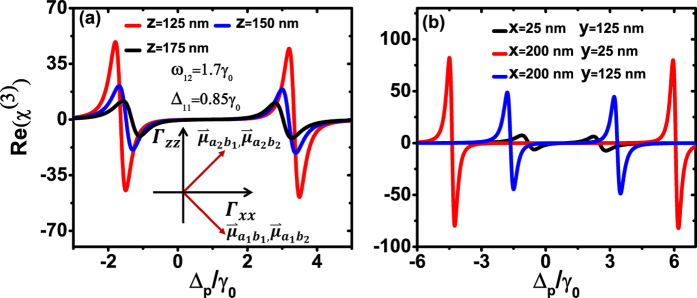
Modified Kerr nonlinearity in a resonant plasmon nanocavity. Kerr nonlinearity Re(*χ*^(3)^) of the double-Λ type system as a function of the probe detuning Δ_*p*_ for (**a**) different distances away from the nanocavity for *z* = 125, 150, 175 nm, with *x* = 200 nm, *y* = 125 nm and (**b**) different locations on the *xy*-plane of *z* = 125 nm. The inset of the (**a**) indicates the direction of the two pairs vertical dipole moments. The Rabi frequency Ω_*c*_ is normalized by 1/5 of amplitude of the electric fields. Other parameters are *ω*_12_ = 1.7*γ*_0_, Δ_11_ = 0.85*γ*_0_, and Rabi frequency Ω_*p*_ = 0.001*γ*_0_.

Finally, we put the quantum emitter into the near field region of designed nanostructure. Letting the *x* axis be the bisector of the two pairs of dipole moments, by varying the distance from *z* = 125 nm to *z* = 175 nm with the fixed *x*, *y* coordinates (*x* = 200 nm, *y* = 125 nm), it is found that the closer to the nanosturcture the bigger Kerr nonlinearity can be obtained due to the larger anisotropic Purcell factors and stronger near field (Fig. 6(a)). Then, we choose three positions *x* = 25 nm, *y* = 125 nm (black curve), *x* = 200 nm, *y* = 25 nm (red curve), *x* = 200 nm, *y* = 125 nm (blue curve) in the *xy* plane with the distance of 75 nm away from the surface of plasmonic nanocavity. As shown in Fig. 6(b), the Kerr nonlinearity are very sensitive to the location of quantum system relative to the custom-designed plasmon structures. It is noticed that, the study of the Kerr nonlinearity influenced by the distance between the quantum system and the plasmonic nanostructure also have been discussed before^41^, but with different mechanism. Using the plasmonic nanostructures’ strong subwavelength near field to trap and manipulate the atoms has been proposed recently^49^,^50^. Although the stability and the accuracy of the atomic position are still need to be increased, our plasmonic nanocavity which offers the near field and suitable Purcell factors within hundreds of nanometers has the possibility to trap the atoms with the current techniques. In addition to the hyperfine structure of the alkali metal atom, the dual CdSe/ZnS/CdSe nanocrystals can be treated as another potential candidate for a four-level system^51^,^52^. To sum up, the anisotropy of Purcell factors and local field enhancement near the resonant plasmonic nanocavity allow for the nanoscale control of enhanced Kerr nonlinearity.

## Conclusion

In summary, we have theoretically investigated the enhanced Kerr nonlinearity of the four-level double-Λ quantum system in the resonant plasmon nanocavity. Using the SGC, we have demonstrated the mechanism of the Kerr nonlinearity modification via anisotropic purcell factors with both vertical and parallel dipole moments. We have also realized the enhanced Kerr nonlinearity at the nanoscale in the combined system composed of the quantum system and custom-designed resonant plasmon nanocavity. This research offers the possibility to utilize the plasmonics nanostructure with the quantum system as a novel quantum nonlinear platform. Such controllable Kerr nonlinearity may be realized by the state-of-the-art nanotechnics and it may have potential applications in the all-optical switches^53^, quantum logic gates^33^, as well as other nanophotonic nonlinear devices.

## Methods

Using the Weisskopf-Wigner theory of spontaneous emission, the systematic equations of motion for the density matrix in the interaction picture involving the cross damping can be derived as follows^29^:


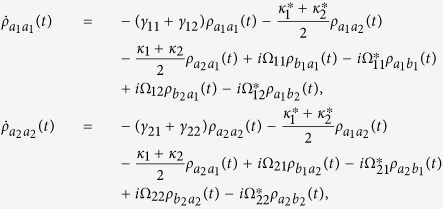



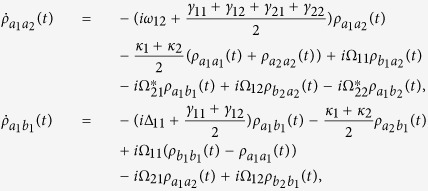



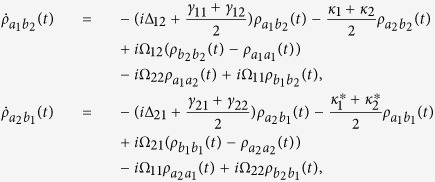



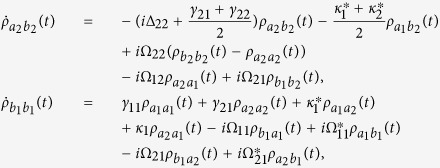



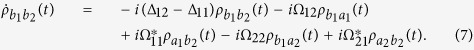


The above equations are constrained by 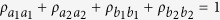 and 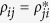. If *y* axis is allowed to be the quantum axis, we use the Γ_*xx*_ and Γ_*zz*_ to denote the decay rates along the *x* and *z* directions and the *θ*_*ij*_ to be the intersection angles between *μ*_*ij*_ and *x* axis. The dissipation term in the anisotropic vacuum can be described by 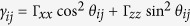 and *κ*_*j*_ = Γ_*xx*_cos*θ*_1*j*_cos*θ*_2*j*_ + Γ_*zz*_sin*θ*_1*j*_sin*θ*_2*j*_, for *i*, *j* = 1, 2, where 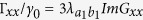, 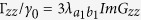, and 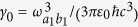 is the decay rate in a vacuum. *G*_*ββ*_ with *β* = *x*, *y*, *z* are represent the Green’s tensor coefficients. In particular, the condition that Γ_*xx*_ = Γ_*zz*_ stands for the isotropic vacuum.

The Green’s tensor coefficients and near field in our designed plasmonic nanostructure are obtained by the Green’s tensor method, which can be used to deal with the arbitrary shaped subwavelength structure^45^,^46^. We consider a subwavelength clusters with the dielectric tensor *ε*(*r*,*ω*) embedding in an infinite homogeneous bulk material with *ε*_0_(*ω*). With the expression of the Green’s tensor in three-dimensional system:


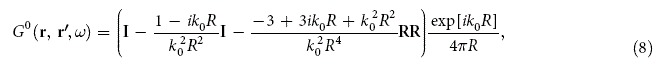


where *R* = |**R**| = |**r** − **r′**| and 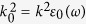, the electric field *E*(**r**) at any point **r** can be given by the Lippmann-Schwinger equation:


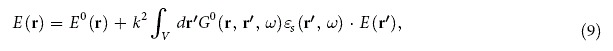


where *V* denotes the clusters subspace, *ε*_*s*_(*r*,*ω*) = *ε*(*r*,*ω*) − *ε*_0_(*ω*). The needed Green’s tensor coefficients can be derived from 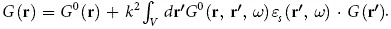.

## Additional Information

**How to cite this article**: Chen, H. *et al*. Nanoscale Kerr Nonlinearity Enhancement Using Spontaneously Generated Coherence in Plasmonic Nanocavity. *Sci. Rep*. **5**, 18315; doi: 10.1038/srep18315 (2015).
